# Bone Mineral Density-Associated Polymorphisms Are Associated with Obesity-Related Traits in Korean Adults in a Sex-Dependent Manner

**DOI:** 10.1371/journal.pone.0053013

**Published:** 2012-12-27

**Authors:** Seongwon Cha, Hyunjoo Yu, Jong Yeol Kim

**Affiliations:** 1 Constitutional Medicine and Diagnosis Research Group, Medical Research Division, Korea Institute of Oriental Medicine (KIOM), Daejeon, Republic of Korea; 2 Korea Institute of Oriental Medicine (KIOM), Daejeon, Republic of Korea; Inserm U606 and University Paris Diderot, France

## Abstract

Obesity and osteoporosis share common physiological factors, including the presence of atherosclerosis, a risk factor for cardiometabolic disease, as well as a common progenitor that differentiates into both adipocytes and osteoblasts. Among the 23 polymorphisms associated with bone mineral density (BMD) in recent genome-wide association studies (GWASs), an *Osterix* polymorphism has been identified and associated with childhood obesity in girls. Therefore, we focused on elucidating polymorphisms associated with adulthood obesity in a sex-dependent manner among the previously published BMD-associated polymorphisms from GWASs. We performed 2 screenings of 18 BMD-associated polymorphisms for obesity-related traits in 2,362 adults aged >20 years. We excluded 13 polymorphisms showing deviations from Hardy–Weinberg equilibrium or no association with obesity-related traits (body mass index, waist circumference (WC), and waist-to-hip ratio). Among 5 selected polymorphisms (rs9594738 of *RANKL*, rs17066364 of *NUFIP1*, rs7227401 of *OSBPL1A*, and rs1856057 and rs2982573 of *ESR1*) analyzed, 2 polymorphisms (rs9594738 and rs17066364) were associated with obesity-related traits. We found sex-dependent associations such that the 4 polymorphisms (excluding rs9594738 of *RANKL*) were associated with abdominal traits such as WC and waist-to-hip ratio only in men. In addition, when the combined genetic risk score (GRS) for WC increase was calculated with 4 SNPs (rs9594738, rs17066364, rs7227401, and rs1856057) exhibiting similar trends for both sexes, the magnitude of the GRS effect for the WC increase was larger in men than in women (effect size = 0.856 cm, *P* = 0.0000452 for men; effect size = 0.598 cm, *P* = 0.00228 for women). In summary, we found 4 polymorphisms, previously related to osteoporosis, to be associated to obesity-related traits in a sex-dependent manner in Korean adults, particularly in men.

## Introduction

Public health problems associated with obesity and osteoporosis share common genetic and environmental factors. Increased body weight strengthens bone, which may decrease osteoporosis risk by increasing bone mineral density (BMD), a well-known indicator for osteoporosis [1], [2]. Recent reports, however, have suggested that the accumulation of abdominal fat mass, regardless of body mass index (BMI), may increase predisposition to osteoporosis risk through BMD reduction [3]–[5]. Therefore, while weight gain by the increase in lean body mass may have a beneficial influence on bone health (mechanical burden), weight gain resulting from increased body fat mass (largely abdominal) may adversely influence bone formation (physiological effect).

Both obesity (especially abdominal obesity) and osteoporosis are physiological risk factors for cardiometabolic diseases. Cardiometabolic disease has been reported to increase osteoporotic risk [6]–[9] and vice versa [7], [9]–[12]. Visceral fat mass may play a critical role in the production of free fatty acids through lipolysis in the adipose tissue, which triggers negative effects on blood vessels, resulting in atherosclerosis [13]–[15]. The presence of calcified plaques in the aorta is strongly related to progressive bone loss [8], [16], [17]. Leptin, a hormone affecting appetite and obesity, may also influence bone mass, although it is not clear whether leptin is negatively associated with bone mass [18], [19]. In addition, both adipocytes and osteoblasts can be derived from a common progenitor, the mesenchymal stem cell, and the balanced differentiation of these cell types may be partially regulated by leptin [20]. These data provide strong evidence of a link between obesity and osteoporosis.

Common genetic variants harboring dual effects on the development of obesity and osteoporosis have been reported. Single nucleotide polymorphisms (SNPs) in the fat mass and obesity–associated (*FTO*) gene, known to influence the risk of obesity, have also been associated with BMD in Chinese populations, based on a recent study reporting lower BMD in mice lacking *Fto* [21]. In addition, the SNPs in the intron of SRY-box 6 gene have been reported to influence both obesity and osteoporosis phenotypes in men [22].

Moreover, among 23 BMD-associated SNPs, which have been recently discovered from several confirmative genome-wide association studies (GWASs), the SNP of the *Osterix* gene influencing bone development has been associated with childhood obesity in women [23]. Since the effects of obesity on fractures may differ between children and adults [24], it is important to determine the genetic effects of BMD-associated SNPs on adulthood obesity. Therefore, we focused on detecting SNPs harboring obesity risk in Korean adults among BMD-associated SNPs previously identified from GWASs [25]–[30]. We also focused on abdominal traits, because a previous study with Korean subjects reported that waist circumference (WC) is associated with bone mineral content independent of total fat mass [5]. As the incidences of osteoporosis and obesity depend on sex, and the effect of the *Osterix* variant is sex-specific, we also estimated sex-dependent risks (individual and combined) of BMD-associated SNPs on obesity.

## Materials and Methods

### Subjects

We recruited 2,362 adults (age >20 years) from 25 oriental medical clinics in Korea over a 5-year period commencing in 2006 as part of the Korea Constitution Multicenter Study. Of these, 975 subjects reported previous diagnosis of hypertension, stroke, dyslipidemia, and/or diabetes, which are known to be correlated with obesity. The remaining recruited subjects were healthy, had subclinical symptoms, or had minor symptoms with no direct relationship to obesity, such as headaches, indigestion, colds, and joint pains. All subjects provided written informed consent to participate in the study, and the study was approved by the Institutional Review Board of the Korea Institute of Oriental Medicine.

WC was measured at the umbilical level, and hip circumference was measured at the level of the widest points around the buttocks. Waist-to-hip ratio (WHR) was calculated by dividing WC by hip circumference. The characteristics of the recruited subjects are presented in Table 1.

**Table 1:** Characteristics of recruited Korean adults.

Characteristic	All (n = 2,362)	Men (n = 915)	Women (n = 1,447)
Age (y)	49.22±14.42	49.22±14.02	49.20±14.87
Body mass index (kg/m^2^)	23.59±3.29	24.17±3.18	23.35±3.41
Waist circumference (cm)	84.41±9.53	87.53±8.47	82.75±9.89
Waist-to-hip ratio	0.90±0.07	0.93±0.06	0.89±0.08
Total cholesterol (mmol/L)	4.86±0.91	4.78±0.89	4.90±0.90
HDL cholesterol (mmol/L)	1.20±0.31	1.07±0.26	1.27±0.31
LDL cholesterol (mmol/L)	2.81±0.80	2.80±0.78	2.82±0.79
Triglyceride (mmol/L)	1.46±0.96	1.72±1.09	1.32±0.88

Data are given as mean ± standard deviation (SD).

### Selection and genotyping of BMD-associated SNPs

We selected 18 BMD-associated SNPs among the polymorphisms reported in previous GWASs [25]–[30]. First, we selected 1 SNP among the SNPs (minor allele frequency ≥0.05) in tight linkage disequilibrium (r^2^≥0.80 in CHB+JPT HapMap populations) within the same gene locus. Second, we replaced the non-existent SNPs in the format of Affymetrix Genome-Wide Human SNP array 5.0 (Affymetrix, Santa Clara, CA) into the proxy SNPs (r^2^ = 1 in CHB+JPT HapMap populations) in the platform of the Affymetrix SNP array 5.0 in order to select the candidate SNPs associated with obesity-related traits (BMI, WC, and WHR) through *in silico* analysis of the data from 714 subjects already genotyped using the SNP array [31]. The polymorphism (rs2016266) of *Osterix* was excluded however, since it was impossible to find a proxy resident in the format of Affymetrix SNP array 5.0. We screened 18 BMD-associated SNPs for associations with obesity-related traits in 2 stages with 2,362 subjects: the first stage involved association screening with 714 subjects (331 men and 383 women), and the second stage involved 1,648 subjects (584 men and 1,064 women). There were no discriminating criteria between 714 subjects and 1,648 subjects, except that the former had been genotyped with Affymetrix SNP array 5.0. After the 2 screening stages, the polymorphisms showing no associations with obesity-related traits (BMI, WC, and WHR) or deviations from Hardy–Weinberg equilibrium (*P*<0.05) were removed such that 13 SNPs (11 SNPs after the first screening and 2 SNPs after the second screening) were excluded in the following analyses (Table S1). That is, we performed the association analyses for obesity-related traits with 5 SNPs in a total of 2,362 subjects.

The genotypes of the 18 BMD-associated SNPs in 714 subjects were determined using the Affymetrix SNP array 5.0. The genotypes of the 7 SNPs in 1,648 subjects were determined by a genotyping method using unlabeled oligonucleotide probes (UOP) on a polymorphic nucleotide [32] (). The UOP was made to span each polymorphism by enabling it to form a perfectly matched duplex with 1 allele. The genomic template containing the SNP site was amplified by polymerase chain reaction (PCR) with a set of primers for each SNP site (Table S1). An aliquot of the PCR amplicon including the SNP site was diluted in a solution containing 1 µM UOP, 5 µM SYTO 9 (Invitrogen, Carlsbad, CA), 12.5 mM EDTA, and 10 mM Tris (pH 8.0). The DNAs in the UOP sample sequentially underwent denaturation (95°C for 5 s), annealing (60°C for 1 min), and melting with a gradual increase to 74°C at a rate of 1°C/s, while the fluorescence emission was read using the LightCycler® 480 instrument (Roche, Indianapolis, IN). The genotype of the polymorphism was determined from 3 melting patterns of the UOP (major homozygote, heterozygote, and minor homozygote). The success rates and concordance rates for genotyping using UOP were >99% and >98%, respectively (Table S1). In the Korean subjects, the genotypes of the 7 polymorphisms were in Hardy–Weinberg equilibrium (*P*>0.05).

### Statistics

We used a chi-squared test to determine whether the polymorphisms were in Hardy–Weinberg equilibrium in the population. We performed multiple linear regression analyses for BMI, WC, and WHR after adjusting for age, sex, physical activity (3 categories), daily food intake (3 categories), history of hypertension, stroke, dyslipidemia, and/or diabetes, and/or menopausal status (in women).

The genetic risk score (GRS) for WC increase was calculated in populations of both sexes with 4 SNPs (rs9594738, rs17066364, rs7227401, and rs1856057), which individually exhibited similar association trends in both men and women, considering the direction of the effect of each SNP on WC. The weighted GRS in each individual was calculated by the sum of the weighted risk score of the 4 SNPs (calculated by multiplying the number of risk alleles by each β-coefficient from the current linear regression analyses for WC) divided by a theoretical maximum sum of the weighted risk scores (8.12 for men and 4.9 for women) of 4 SNPs and multiplied by 8 [33]. The individuals (5 men and 14 women) with missing genotypes for the 4 SNPs were excluded from the GRS analyses. A *p*-value of <0.05 was considered nominally significant, and the significance for multiple comparisons was further adjusted with a false discovery rate (FDR) (significance: *p*<0.05 and FDR<0.1), suggested by Benjamini and Hochberg [34]. All regression analyses were performed using R, version 2.10.1 (http://www.r-project.org/).

## Results

### Individual association of BMD-associated SNPs with obesity-related traits

We performed analyses related to the genetic effects of 5 BMD-associated SNPs on BMI, WC, and WHR, as representatives of obesity-related traits in 2,362 Korean adults. Our findings indicated that 2 SNPs (rs9594738 of receptor activator of nuclear factor-*k*B ligand (*RANKL*) and rs17066364 of nuclear fragile X mental retardation protein interacting protein 1 (*NUFIP1*)) were significantly associated with BMI and abdominal traits (WC for rs9594738; WHR for rs17066364) (Table 2). The rs7227401 of oxysterol binding protein-like 1A (*OSBPL1A*) and rs1856057 of estrogen receptor 1 (*ESR1*) were nominally associated with BMI and abdominal traits, respectively.

**Table 2:** Associations of individual BMD-associated SNPs with obesity-related traits.

			All (n = 2,362)			Men (n = 915)			Women (n = 1,447)		
SNP (Gene)	Allele (MAF)	Phenotype	n	Effect size (95% CI)^a^	*P* a (FDR)	n	Effect size (95% CI)^a^	*P* a (FDR)	n	Effect size (95% CI)^a^	*P* a (FDR)
rs9594738	C>T	BMI	2,357	**−0.466 (−0.779, −0.153)**	**0.00348 (0.092)**	913	−0.254 (−0.759, 0.252)	0.326 (0.526)	1, 444	−0.605 (−0.986, −0.196)	0.00288 (0.112)
(*RANKL*)	(8.66)	WC		**−1.11 (−1.95, −0.275)**	**0.00928 (0.092)**		−0.615 (−1.94, 0.705)	0.360 (0.529)		−1.49 (−2.56, −0.428)	0.00602 (0.117)
		WHR		−0.00502 (−0.0111, 0.00106)	0.106 (0.412)		0.000674 (−0.00844, 0.00979)	0.884 (0.932)		−0.00925 (−0.0172, −0.00133)	0.0221 (0.269)
rs17066364	G>C	BMI	2,356	**0.351 (0.0863, 0.617)**	**0.00942 (0.092)**	914	0.280 (−0.149, 0.709)	0.200 (0.504)	1, 442	0.381 (0.0423, 0.715)	0.0276 (0.269)
(*NUFIP1*)	(12.6)	WC		0.721 (0.00815, 1.43)	0.0474 (0.231)		0.754 (−0.365, 1.87)	0.186 (0.504)		0.655 (−0.251, 1.56)	0.156 (0.835)
		WHR		**0.00742 (0.00227, 0.0126)**	**0.00475 (0.092)**		**0.0102 (0.00255, 0.0179)**	**0.0091 (0.089)**		0.00523 (−0.00151, 0.0119)	0.127 (0.835)
rs7227401	G>T	BMI	2,353	0.304 (0.0302, 0.577)	0.0295 (0.171)	913	0.484 (0.0506, 0.918)	0.0287 (0.186)	1, 440	0.213 (−0.154, 0.546)	0.235 (0.835)
(*OSBPL1A*)	(12.0)	WC		0.658 (−0.0761, 1.39)	0.0789 (0.342)		**1.51 (0.383, 2.64)**	**0.00874 (0.089)**		0.202 (−0.739, 1.14)	0.673 (0.950)
		WHR		0.000676 (−0.00463, 0.00598)	0.803 (0.988)		0.00458 (−0.00323, 0.0124)	0.2501 (0.526)		−0.00109 (−0.00808, 0.00590)	0.760 (0.970)
rs1856057	T>C	BMI	2,358	−0.0953 (−0.292, 0.101)	0.341 (0.844)	914	−0.269 (−0.580, 0.0404)	0.0881 (0.344)	1, 444	0.0408 (−0.199, 0.305)	0.752 (0.970)
(*ESR1*)	(26.8)	WC		−0.581 (−1.11, −0.0543)	0.0307 (0.171)		**−1.17 (−1.99, −0.372)**	**0.00426 (0.083)**		−0.100 (−0.779, 0.579)	0.771 (0.970)
		WHR		−0.00479 (−0.00860, −0.000985)	0.0136 (0.107)		−0.00574 (−0.0113, −0.000164)	0.0436 (0.226)		−0.00354 (−0.00859, 0.00151)	0.168 (0.835)
rs2982573	A>G	BMI	2,357	−0.0391 (−0.319, 0.241)	0.784 (0.988)	912	−0.440 (−0.885, 0.00424)	0.0522 (0.226)	1, 445	0.208 (−0.149, 0.568)	0.258 (0.840)
(*ESR1*)	(11.0)	WC		−0.566 (−1.31, 0.186)	0.140 (0.497)		**−1.88 (−3.04, −0.724)**	**0.00146 (0.057)**		0.282 (−0.682, 1.25)	0.566 (0.950)
		WHR		−0.00301 (−0.00845, 0.00243)	0.277 (0.773)		−0.00628 (−0.0143, 0.00172)	0.123 (0.439)		−0.000801 (−0.00798, 0.00637)	0.826 (0.970)

Abbreviations: MAF, minor allele frequency; CI, confidence interval; FDR, false discovery rate; BMI, body mass index; WC, waist circumference; WHR, waist-to-hip ratio.

a Linear regression: adjustment for age, sex, physical activity (3 categories), daily food intake (3 categories), chronic disease status, and/or post-menopause (Boldface indicates a significant association at *p*<0.05 and FDR<0.1.).

When we examined the association signals for obesity-related traits of the 5 SNPs in populations of both sexes, we found that 4 SNPs (rs17066364, rs7227401, rs1856057, and rs2982573) were significantly associated only in men (Table 2). The effects of these 4 SNPs, especially on abdominal traits, were enriched more significantly in men. Although the effect of SNP rs9594738 on obesity-related traits appeared to be enriched more significantly in women, the significance was diminished after adjustment for multiple testing at FDR = 1.0 (Table 2).

### Combined GRS assessment for WC increase in a sex-dependent manner

Four SNPs (rs9594738, rs17066364, rs7227401, and rs1856057) were chosen in order to calculate the combined GRS on WC increase in men and women independently. We did not use rs2982573 to estimate the GRS, because the SNP was linked to rs1856057 within the same linkage disequilibrium block (r^2^ = 0.46, D′ = 0.95) in HapMap Han Chinese and Japanese populations (HapMap-HCB and JPT) via the Haploview program (version 4.2) [35]. Considering the GRS as a continuous variable, the magnitude of the GRS effect for the WC increase was larger in men than in women (WC, effect size = 0.856 cm, *P* = 0.0000452 for men; effect size = 0.598 cm, *P* = 0.00228 for women). On comparing the subjects in the highest quartile (over 75th percentile) of the GRS and those in the lowest quartile (below 25th percentile) of the GRS, the sex-dependent effects of GRS on WC increase were obvious. Men in the highest quartile of the GRS in comparison with those in the lowest quartile of the GRS showed an increase in WC of 3.01 cm (Table 3). However, the difference in women was not significant. These sex-dependent results might be due to stronger association signals of each SNP in men.

**Table 3:** Assessment of combined GRS for WC increase in populations of both sexes.

GRS feature^a^	Subgroup^b^	n	Median GRS^a^ (range)	Effect size (95% CI)^c^	*P* c
**Men**					
Continuous	–	910	3.55 (0.61–6.65)	0.856 (0.446, 1.27)	0.0000452
Dichotomous	Over Q3	227	5.03 (4.00–6.65)	3.01 (1.58, 4.44)	0.000430
	Below Q1	267	2.38 (0.61–2.38)		
**Women**					
Continuous	–	1,433	5.19 (0.00–7.67)	0.598 (0.214, 0.983)	0.00228
Dichotomous	Over Q3	407	6.11 (5.52–7.67)	1.03 (−0.0122, 2.08)	0.0527
	Below Q1	553	4.87 (0.00–5.03)		

Abbreviations: GRS, genetic risk score; WC, waist circumference; CI, confidence interval.

a Polymorphisms used in GRS: rs9594738, rs17066364, rs7227401, and rs1856057.

b Subgroups: below Q1, subjects in the lowest quartile of the GRS; over Q3, subjects in the highest quartile of the GRS.

c Linear regression: adjustment for age, physical activity (3 categories), daily food intake (3 categories), chronic disease status, and/or post-menopause.

## Discussion

We found that 2 of 18 BMD-associated SNPs were associated with obesity-related traits (BMI, WC, and/or WHR) in all population groups (Table 2). Sex differences in the associations with obesity-related traits have been observed [36]; accordingly, in our study, we found that 4 SNPs (rs17066364, rs7227401, rs1856057, and rs2982573) were significantly associated with obesity-related traits in men (after adjusting with FDR), whereas no association signals were observed in women (SNP rs9594738 showed a marginal association) (Table 2). In the 2 SNPs (rs17066364 and rs2982573), the minor alleles previously reported to be associated with increased BMD were associated with decreased obesity-related traits in men, and vice versa. In case of rs7227401, although the minor allele associated with increased WC in men is known to be associated with increased femur neck width [27], it may also be associated with decreased BMD due to the negative correlation [37] between femur neck width and femur BMD. These phenomena correspond with the results of previous studies indicating that increased fat mass (correlated with BMI, WC, and WHR) increases susceptibility to osteoporosis by lowering the BMD [5]. The observed lack of changes in obesity-related traits in women with BMD-associated SNPs may be due to the relatively small genetic effects of the SNPs in this population, or it may indicate environmental factors that obscure the real genetic effects. In fact, it has been shown that pregnancy, lactation, and/or menopause increase the propensity for osteoporosis by lowering BMD and obesity [38], [39]. In our study, we controlled the postmenopausal status in regression analyses, but it was impossible to adjust for the influences of pregnancy and lactation. Conversely, men may be relatively less susceptible to environmental changes (e.g., hormonal changes) compared to women.

Functional assumptions for the genetic loci using previous reports may help in understanding the actions of the polymorphisms on bone metabolism and obesity, but these assumptions cannot replace scientific evidence. Serum levels of RANKL encoded by tumor necrosis factor superfamily 11 gene, of which rs9594738 is located 185 kb upstream, is reduced by omentin-1 [40]. Omentin-1 is inversely related to obesity [41]. It has also been reported that mice fed with a corn oil–enriched diet exhibited increases in visceral and total body fat mass, as well as incremental increases in adipocyte-specific peroxisome proliferator-activated receptor gamma protein, bone marrow adiposity, and osteoclast-specific RANKL protein expression [42]. Therefore, these results suggest that RANKL may be correlated with bone metabolism, as well as with obesity.

The *NUFIP1* containing rs17066364 SNP in an intron interacts with a fragile X mental retardation protein (*FMRP*) [43]. Boys with fragile X syndrome exhibit a predisposition to increased obesity [44], although no linkage has been established between *NUFIP1* and obesity.

The polymorphism rs7227401 is located in an intron of *OSBPL1A*, which is believed to exhibit functions similar to those of oxysterol-binding protein (*OSBP*) [45]. Interestingly, OSBP is known to increase the mRNA levels of sterol regulatory element binding-protein-1c and insulin-induced gene 1 involved in adipogenesis [46]. Therefore, *OSBPL1A* might play a role in adipogenesis metabolism.

The rs2982573 SNP is located 1 kb upstream of *ESR1*, while rs1856057 is located in the intron of *ESR1*. The 2 SNPs (rs1999805, perfect linkage with rs1856057; rs2941740, perfect linkage with 2982573) were identified within the same linkage disequilibrium block (r^2^ = 0.46, D′ = 0.95) in HapMap-HCB and JPT. This linkage between 2 SNPs may indicate a similar association with WC in men. However, the reported associations of 2 SNPs with BMD in European descendants appear contradictory [25], [26]. Although no data are currently available directly comparing the associations between WC and the 2 SNPs in subjects of European descent, the differential association for BMD between 2 SNPs may be due to a relatively weak linkage in the HapMap Caucasian population (HapMap-CEU) (r^2^ = 0.32, D′ = 0.76), despite very similar minor allele frequency (0.44 for rs1856057 vs. 0.43 for rs2982573). The associations of *ESR1* variants with obesity-related traits are supported by the association of *Pvu*II polymorphism of *ESR1* with total fat mass in young Chinese men [47] and by the manifestation of obesity and insulin resistance in ER alpha (ESR1)^−/−^ mice [48].

One of the main limitations of this study is the omission of BMD data; therefore, we could not directly compare the association signals for BMD with those for obesity-related traits. However, the BMD-associated SNPs used in this study have been confirmed for BMD association through GWASs and related studies. Another limitation of this study is that we did not directly measure the visceral fat mass showing negative correlation with BMD. Although the measured WC is strongly correlated with visceral fat mass, WC is considered an estimation of both the fat mass and the lean body mass. Therefore, our findings suggest a positive relation between abdominal traits and BMD via BMD-associated SNPs. It would be necessary to measure visceral fat mass by using computed tomography and dual-energy X-ray absorptiometry for more accurate assessment of the genetic effects of those SNPs on obesity.

In conclusion, we estimated BMD-associated SNPs from GWASs for obesity risks, and we identified 2 BMD-associated SNPs showing association with obesity-related traits (BMI, WC, and WHR) in all adult subjects. In addition, the association of 4 BMD-associated SNPs with abdominal traits occurred in a sex-dependent manner, with genetic effects (both individual and combined) of the SNPs stronger in men than in women. It may be possible to prevent the development of these diseases in people susceptible to osteoporosis and obesity by assessing genetic risks in advance using those SNPs harboring dual effects on osteoporosis and obesity in a sex-dependent manner.

## Supporting Information

Table S1 Selection of 5 polymorphisms after 2 screening stages with 18 BMD-associated polymorphisms.
(XLS)
